# Impact of Th-17 Cytokines on the Regulation of Transporters in Human Placental Explants

**DOI:** 10.3390/pharmaceutics13060881

**Published:** 2021-06-15

**Authors:** Kamelia Mirdamadi, Jacinda Kwok, Ori Nevo, Howard Berger, Micheline Piquette-Miller

**Affiliations:** 1 Department of Pharmaceutical Sciences, Leslie Dan Faculty of Pharmacy, University of Toronto, Toronto, ON M5S 3M2, Canada; kamelia.mirdamadi@mail.utoronto.ca (K.M.); jacinda.kwok@mail.utoronto.ca (J.K.); ori.nevo@sunnybrook.ca (O.N.); 2 Maternal-Fetal Medicine, Department of Obstetrics and Gynecology, Sunnybrook Health Sciences Centre, Toronto, ON M4N 3M5, Canada; 3 Maternal-Fetal Medicine, Department of Obstetrics and Gynecology, St. Michael’s Hospital, Toronto, ON M5B 1W8, Canada; bergerh@smh.ca

**Keywords:** Th-17, interleukin 17, cytokines, placenta, drug transporters, explants, inflammation

## Abstract

Activated T helper 17 (Th-17) cytokines play a role in the pathophysiology of autoimmune and infectious diseases. While these diseases affect many women of childbearing age, little is known about the effect of these cytokines on placental transporters. As several pro-inflammatory cytokines impact the expression of ABC and SLC placental transporters, we hypothesized that these transporters may be similarly altered by elevated levels of circulating Th-17 cytokines. Cultured term human villous explants were treated with IL-17A, IL-22, or IL-23, alone or in combination. Samples were analyzed using qRT-PCR and Western blotting. The mRNA expression of OATP2B1 was significantly downregulated in explants by all individual cytokines and combination treatments, while decreased protein expression was seen with IL-23 and combination (*p* < 0.01). Combination treatment decreased the mRNA expression of BCRP and OAT4 but increased that of OCT3 (*p* < 0.01). Decreased accumulation of the OATP substrate, cascade blue, was seen in IL-23-treated choriocarcinoma JAr cells (*p* < 0.01). Elevated Th-17 cytokines, which are seen in infectious and autoimmune diseases, affect the expression and activity of OATP2B1, as well as mRNA expression of placental BCRP, OAT4, and OCT3. This dysregulation could impact the fetal exposure to endogenous and exogenous substrates.

## 1. Introduction

Activated T helper 17 (Th-17) CD4+ cells are major contributors to the pathogenesis of several autoimmune diseases and inflammatory conditions. Several cytokines and transcription factors, such as interleukin (IL)-1β, IL-6, IL-23, TGF-β, and STATs (signal transducer and activators of transcription) are involved in the differentiation of activated Th-17 cells [1,2]. Expression of IL-17A, IL-22, and IL-23R by activated Th-17 cells, increases the production of pro-inflammatory cytokines and chemokines in target tissues, thereby resulting in systemic inflammatory effects [3,4]. Up to 8-fold higher levels of IL-17A and 5-fold higher levels of IL-22 are seen in the serum of patients with autoimmune diseases such as rheumatoid arthritis [5,6], while a 3-fold increase in serum levels of IL-23 has been reported in patients with multiple sclerosis [7]. Recent studies have also reported the involvement of Th-17 CD4+ cell activation and IL-17 release in the pathogenesis of the cytokine storm and severe outcomes associated with COVID-19 [8]. It is recognized that increased systemic production of pro-inflammatory cytokines due to acute or chronic inflammatory conditions can impact the expression and activity of many clinically important drug metabolizing enzymes and membrane transporters [9,10].

Notably, infectious and autoimmune inflammatory conditions affect many women of child-bearing age, and increased incidences of adverse pregnancy outcomes have been reported in these conditions [11,12,13]. The placenta, serving as the major connection between maternal and fetal circulation, expresses several important ATP-binding cassette (ABC) and solute carrier (SLC) transporters [14], which play an important role in nutrient delivery and fetal protection against maternal-borne toxins and xenobiotics. Previous in vivo studies from our laboratory have shown that induction of proinflammatory cytokines following administration of viral and bacterial mimetics to pregnant rats imposes a downregulation of placental transporters, including P-glycoprotein (P-gp), the breast cancer resistance protein (BCRP), and the organic anion transporting polypeptide 2B1 (OATP2B1), which in turn, was associated with changes to maternal and fetal exposure to their substrates [15,16]. Moreover, in vitro treatment of primary trophoblasts with pro-inflammatory cytokines such as tumor necrosis factor (TNF)-α and IL-6 has been reported to alter the expression and activity of several ABC transporters [17]. As such, maternal diseases may alter fetal exposure to exogenous and endogenous substances, through dysregulation of placental transport systems [18].

Although there is ample evidence to support inflammation-mediated changes in placental transporters, it remains unknown whether the Th-17-associated cytokines alter the expression of transporters in the placenta. Given the high prevalence of Th-17-associated infectious and immune disorders that occur in women of childbearing age and their potential implications for pregnancy outcomes, the objective of this study was to investigate the impact of IL-17A, IL-22, and IL-23 on the expression of transporters, using cultured placental term explants. We examined the most abundantly expressed placental drug transporters, including the organic cation transporter 3 (OCT3/SLC22A3), organic anion transporter 4 (OAT4/SLC22A11), organic anion-transporting polypeptide 2B1 (OATP2B1/SLCO2B1), and breast cancer resistance protein (BCRP/ABCG2) [19].

## 2. Materials and Methods

### 2.1. Placental Tissue Culture

Human placental villous explants were prepared from ten term (≥38 weeks) placentas from women undergoing elective caesarean section delivery, without any pregnancy complications such as preeclampsia or fetal growth restriction. Placentas were collected immediately after delivery at St. Michael’s Hospital or Sunnybrook Health Sciences Center (Toronto, ON, Canada). The study was conducted according to the guidelines of the Declaration of Helsinki, and approved by the Ethics Committees of St. Michael’s hospital (protocol code 17-263, date of approval November 2019) and Sunnybrook Health Sciences Center (protocol code 136-2008, date of approval May 2020). Informed consent was obtained from all study participants involved in the study. Explants were cultured within 30 min of delivery, as previously described [20]. In brief, intact cotyledons were excised by blunt dissection, where lipids and blood vessels were removed. Samples were washed with ice-cold PBS, and two pieces of dissected tissue (30–50 mg each) were placed in each well of a 24-well plate (Day 1). Explants were cultured in 1.5 mL of Dulbecco’s Modified Eagle Medium/F12, supplemented with 10% FBS, 0.1% non-essential amino acids (NEAA), 0.1% penicillin/streptomycin/normocin, and 0.1% insulin at 37 °C in 5% CO_2_/air, and allowed to equilibrate for 24 h, prior to treatments. On experimental day 2, media was replaced with fresh media (controls) or fresh media containing IL-17A (0.1 or 1.0 ng/mL) (New England Biolabs, Whitby, ON, Canada), IL-22 (0.1 or 1.0 ng/mL) (Cedarlane, Burlington, ON), or IL-23 (0.1 or 1.0 ng/mL) (Cedarlane, Burlington, ON, Canada), or with a combination (Comb) of the three cytokines (IL-17A + IL-22 + IL-23; 0.1: 0.1: 0.3 ng/mL), and incubated for 24 h (Day 3). Explants were then collected and stored at −80 °C, until analysis. Separate treated and untreated explants were used for mRNA or protein analysis. Media from each well was collected before and after treatment, and stored at −80 °C for lactate dehydrogenase (LDH) and human chorionic gonadotropin (hCG) testing.

### 2.2. Quantitative Real-Time PCR Analysis

The mRNA expression of BCRP, OAT4, OATP2B1, and OCT3 was determined in the treated and untreated (control) explants, using qRT-PCR, as previously described [21]. In brief, RNA was extracted from placental explants using Trizol (Invitrogen, Carlsbad, CA, USA) and total RNA concentrations were quantified using the NanoDrop 1000 spectrometer (Thermo Fisher Scientific. Waltham, MA, USA). RNA (2 µg) was first treated with DNase (Thermo Scientific) and reverse-transcribed (RT) using a high-capacity cDNA RT kit (Applied Bio Systems, Waltham, MA, USA). qRT-PCR was performed using Power SYBR™ Green (Applied Bio Systems, Waltham, MA) with the CFX384 Touch Real-Time PCR System (BIO-RAD Laboratories, ON, Canada). The relative expression of mRNA was determined using the ΔΔCt method normalized to the mean of the housekeeping genes, cyclophilin A, and UBC. All mRNA levels were normalized to the expression of the untreated controls. Primer sequences are listed in Table 1.

### 2.3. Immunodetection

Crude membrane fractions were isolated from 30–50 mg of human placental explants, as previously described [22]. Explant tissues were homogenized in 2 mL of homogenizing buffer containing 0.1 M Tris-HCl, pH 7.5, 3 µL/mL protease inhibitor, and 50 µg/mL PMSF. Another 4 mL of homogenizing buffer was added to the homogenates, which were centrifuged at 2000× *g* for 20 min at 4 °C. The supernatant was collected and centrifuged at 100,000× *g* for 1 h at 4 °C and the resulting pellet was collected. Protein concentrations were measured using the Pierce BCA Protein Assay Kit (Thermo Scientific, Waltham, MA, USA). Crude membrane samples (10–30 µg) in the Laemmli loading buffer were heated at 37 °C for 20 min, separated via 10% SDS-PAGE, transferred to PVDF membranes (Bio-Rad Laboratories, ON, Canada), and incubated overnight at 4 °C, with a primary antibody (OATP2B1; anti-SLCO2B1; 1:500; Abcam, OCT3; anti-SLC22A3 Q306; 1:1000, Bioworlde, St. Louis Park, MN, USA) in 2% non-fat milk in TBST (TRIS-buffered saline-Tween-20). Membranes were then washed with TBST and incubated with an HRP-labelled secondary antibody (anti-mouse; 1:10,000 or anti-rabbit; 1:1000, Millipore Sigma, Oakville, ON, Canada) for 1 h at room temperature. Densitometry was performed using ChemiDoc Imaging System 12003153 (Bio-Rad Laboratories, ON, Canada). For Simple Western detection of BCRP, 5 µg membrane fraction samples were separated using capillary electrophoresis under default run conditions on WES (Protein Simple, San Jose, CA, USA), using primary antibodies for BCRP (BXP-21; 1:500, Santa Cruz) and β-actin. Proteins of interest were detected using the anti-mouse detection kit (#DM-002, Protein Simple) and analyzed using the Compass Software version 3.1 for Simple Western (Protein Simple). All protein levels were normalized to β-actin (AC15; 1:75,000; Millipore Sigma, Oakville, ON, Canada) and expressed relative to the expression of the untreated controls.

### 2.4. Viability Analysis

Tissue viability of explants was determined prior to treatment on Day 2 and 24 h after treatment on Day 3, by measuring Lactate Dehydrogenase (LDH) secretion in the culture media, using a colorimetric LDH detection kit, as per the manufacturer’s instructions (Sigma-Aldrich, Saint Louis, MO, USA). In brief, 25 µL of culture media and 75 µL of the reaction mixture, catalyst, and dye solution were added into a 96-well plate. For low control, 1% BSA (bovine serum albumin) was used; 25 µL of sample and 75 µL of 2% TritonX-100 in assay medium solution was used as high control. Low control, high control, and unknowns were all assayed in triplicates. The plates were then incubated for 30 min at 37 °C and 5% CO_2_, and read at 490 nm and 620 nm (Cytation5, BioTek, Winooski, VT, USA).

Functional endocrine activity of placental explants was examined by measuring the secretion of human chorionic gonadotropin (hCG) into the culture media. Explant production of hCG is reflective of tissue viability [23]. Levels in culture media were measured prior to treatments on Day 2 and 24 h after treatment (Day 3), using an hCG ELISA kit (Sigma-Aldrich, Saint Louis, MO, USA), as per the manufacturer’s instructions. In brief, all standards and samples were added into a 96-well plate in triplicates and incubated for 2.5 h at room temperature. Wells were washed four times using 1% wash buffer and the plates were blotted to remove the wash buffer residuals. A total of 100 µL of biotinylated detection antibody was added to each well and incubated for one hour at room temperature and the wash step was repeated. A total of 100 µL of the HRP-streptavidin solution was added to each well and incubated for 45 min at room temperature. The wash step was repeated and 100 µL of ELISA colorimetric reagent was added to each well and incubated in the dark for 30 min, at room temperature. Plates were washed 4 times, 50 µL of the stop solution was added to each well, and absorbance was immediately measured at 450 nm. The assay’s limit of detection was 50 pg/mL.

### 2.5. Functional Transport Assay

The cascade blue hydrazide (CB) fluorescent dye transport assay, modified from the previously published protocols [24,25], was used to monitor OATP transport activity in IL-23-treated and untreated human choriocarcinoma JAr cells. JAr cells were seeded into 12-well plates and cultured for 48 h at 37 °C, 5% CO_2_. For gene expression analysis, the JAr cells were treated with 0.1 and 1.0 ng/mL IL-23 or media alone for 24 h. The mRNA expression of OATP2B1 was determined by qRT-PCR, as described for explants. For the OATP transport assay, confluent cells were treated with 1.0 ng/mL IL-23 (treated) or media alone (untreated control), for 24 h, before the accumulation assays were performed. On the day of the assay, cell medium was removed, and the cells were washed three times with 1 mL of phosphate-buffered saline (PBS). Transport experiments were conducted in Hank’s buffer with the pH adjusted to 6.5, using 10 N NaOH. Cells were pre-incubated for 1 h in the uptake buffer. Cells were then incubated with 10 µM fluorescent CB for 30 min at 37 °C and accumulation was stopped by the addition of 1 mL ice-cold PBS. The supernatant was rapidly aspirated, cells were washed three times with 1 mL ice-cold PBS, and 500 μL PBS was added. Fluorescence was measured at room temperature using the Cytation5 cell imager (BioTeK, VT, USA) at wavelengths of 400 and 419 nm for excitation and emission, respectively. Concentrations of the substrate (CB), the inhibitor (CsA), pH, and accumulation times were optimized in the pilot experiments. Cell viability following treatment was assessed using the methylthiazol tetrazolium (MTT) assay, as previously described [26]. Following treatment, 5 mg/mL MTT solution was added to cells and incubated for 2 h at 37 °C, 5% CO_2_. Absorbance was measured at 570 nm, using the Cytation 5 cell imager (BioTek, Winooski, VT, USA).

### 2.6. Statistical Analysis

Statistical analyses were performed using Prism 5.3 (GraphPad Software; San Diego, CA, USA). One-way ANOVA was used to compare the variance between cytokine treatment groups with subsequent analysis using Dunnet’s multiple comparisons test. Two-tailed *t*-test was used to compare mean differences between the controls and combination treatments. All data are presented as mean ± standard error of the mean (SEM). Significance was defined as *p* < 0.05.

## 3. Results

### 3.1. Effect of Th-17 Cytokines on Transporter Expression

**Figure 1:**
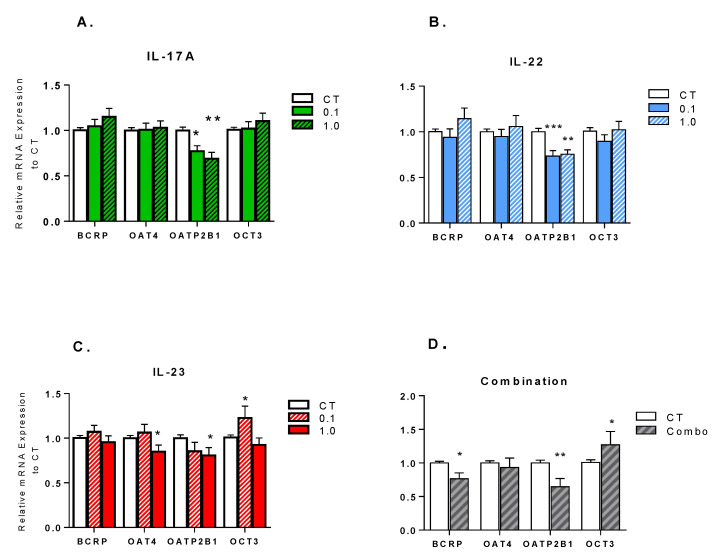
Impact of (**A**) IL-17A; (**B**) IL-22; (**C**) IL-23; or (**D**) IL-17A/IL-22/IL-23 combination treatment on the mRNA expression of transporters, in the term placental villous explants. Explants were treated with 0.1 ng/mL or 1 ng/mL of cytokine, combination (IL-17:IL22:IL-23; 0.1:0.1:0.3 ng/mL), or media (control, CT) for 24 h. Expression was determined by qPCR and normalized to the housekeeping genes. Data are normalized to the untreated control explants and presented as mean ± SEM. All experiments were performed in triplicates from 8–10 different placentas. Means were compared by ANOVA, followed by Dunnett’s post-hoc analysis. * *p* < 0.05, ** *p* < 0.01, *** *p* < 0.001 vs. control.

As compared to controls, 24-h treatment of explants with individual cytokines significantly downregulated the mRNA levels of OATP2B1 (Figure 1A–C). Treatment of explants with IL-23, but not with IL-17A or IL-22, significantly decreased mRNA levels of OAT4 (*p* < 0.05), while levels of OCT3 (*p* < 0.05) were significantly increased. The mRNA expression of BCRP was not significantly altered by any individual cytokine treatment. Combination treatment with these cytokines significantly downregulated the mRNA expression of BCRP and OATP2B1 (*p* < 0.01), and significantly increased the mRNA levels of OCT3 (Figure 1D).

**Figure 2:**
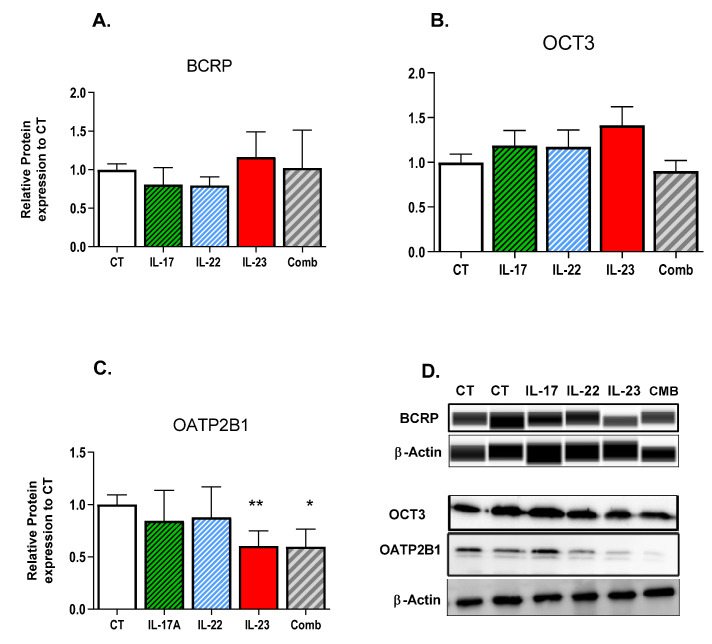
Impact of individual or combined treatments with IL-17, IL-22, and IL-23 on the protein expression of (**A**) BCRP; (**B**) OCT3; and (**C**) OATP2B1 in term placental villous explants. (**D**) Representative immunodetection. Placental explants were treated with 1 ng/mL of cytokine, combination (17: 22: 23 = 0.1:0.1:0.3 ng/mL), or media (control, CT) for 24 h. Protein expression was determined by Western blotting or Simple Western and normalized to β-actin. Data are normalized to control and presented as mean ± SEM, N = 5–7 different placenta. Means were compared by one-way ANOVA, followed by Dunnett’s post-hoc analysis. * *p* < 0.05, ** *p* < 0.01 vs. control.

At the protein level, OATP2B1 was significantly downregulated in explants treated with IL-23 or combined cytokines (*p* < 0.05) (Figure 2C). Protein levels of BCRP and OCT3 were not affected by either individual or combined cytokine treatments (Figure 2A,B).

### 3.2. Effect of IL-23 on OATP Transport Activity

**Figure 3:**
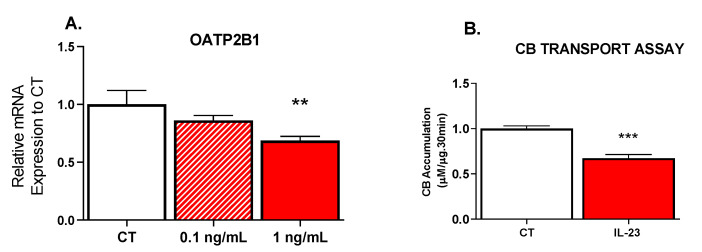
Effect of IL-23 on the (**A**) mRNA expression of OATP2B1 and (**B**) intracellular accumulation of Cascade Blue (CB) in JAr cells. JAr cells were treated with IL-23 or media (CT) for 24 h, and either collected for RNA or used for the CB transport assay, as described in methods. Three or four replicates were run in three separated experiments (*n* = 3). Data are presented as mean ± SEM. ** *p* < 0.01, *** *p* < 0.001.

Consistent with the downregulation of OATP2B1 mRNA, pre-treatment of JAr cells with IL-23 for 24 h, decreased the intracellular uptake/accumulation of CB by approximately 30% (Figure 3A). Cell viability was not impacted by treatments, as measured by the MTT assay (data not shown).

### 3.3. Effect of Th-17 Cytokines on Tissue Viability and Inflammatory Response

**Figure 4:**
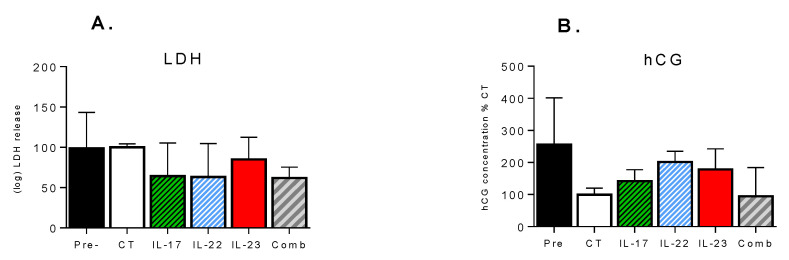
Effect of cytokine treatments on the viability of cultured placental explants, as measured by (**A**) Lactate Dehydrogenase (LDH) release and (**B**) human chorionic gonadotropin (hCG) secretion, over the 24 h treatment period. LDH and hCG were measured in the culture media collected from 6–8 explants and 3–4 explants, respectively. Pretreatment (Pre) levels are shown. Data are normalized to the untreated controls (CT) and are presented as mean ± SEM. No significant differences were detected.

LDH testing revealed that cytokine treatments were not cytotoxic to tissue explants, as LDH levels in the culture media of the treated explants on Day 3 were not significantly different from levels seen before treatments on Day 2 nor from Day 3 controls. Moreover, LDH levels in the cultured media of the untreated controls on Day 3 were not significantly different from the levels on Day 2. Treatment of explants with either individual or combined cytokines did not significantly affect the hCG release between Day 2 or Day 3, and was not significantly different from the untreated controls (Figure 4B). A 24-h treatment of explants with individual cytokines, as well as with combination treatment, did not affect the transcript levels of IL-6 and TNF-α (data not shown).

## 4. Discussion

Although several studies have demonstrated dysregulation of placental drug transporters due to inflammation-mediated induction of Th-1 cytokines [21,27], the effect of Th-17-associated cytokines has not been explored. While Th-17 cytokines, including IL-17A, IL-22, and IL-23, are involved in maintaining the health of a pregnancy, numerous inflammatory or immune-related conditions are associated with a pronounced elevation of these cytokines and may adversely affect pregnancy and neonatal outcomes [28,29]. For example, while maternal serum IL-17A levels of 1–2 pg/mL are considered normal, levels of up to 10–30 pg/mL are seen in pregnancies complicated with systemic lupus erythematosus (SLE) [30]. Significantly increased IL-23 concentrations of up to 1.38 ng/mL have also been reported in SLE patients [31]. In this study, we demonstrated that treatment of healthy term human placental explants with IL-17A, IL-22, and IL-23, and their combination, imposed significant downregulations in the mRNA and in protein expression of OATP2B1. Th17 cytokines are known to have synergistic effects in combination, therefore, their effects may be more apparent when combined as compared to effects seen as individual cytokines [2,28]. Indeed, treatments with clinically relevant combinations of these cytokines were also found to alter the transcript but not the protein levels of BCRP and OCT3.

Previous clinical studies from our laboratory have demonstrated that bacterial infection such as chorioamnionitis, reduces the protein expression of OATP2B1 in placenta, to 50% of that seen in the placentas obtained from healthy pregnancies [22], while a 2-fold increase in the expression of OATP2B1 was seen in placentas obtained from pregnancies complicated with pre-eclampsia [21]. A pronounced decrease in transcript levels was also seen in placentas obtained from HIV (+) women [32]. Treatment of human choriocarcinoma JAr cells with IL-23 was also found to decrease the OATP2B1 expression and this was associated with a significant reduction in the cellular uptake and accumulation of the OATP substrate cascade blue (CB). CB has been shown to be a good fluorophore to study OATP function, as it has a very low passive membrane permeability and cellular uptake is mediated via OATP2B1, which is highly expressed in the placenta, as well as by the liver-specific OATP1B1 [24]. Within the placenta and JAr cells, OATP2B1 is the only OATP transporter to be expressed to an appreciable extent. It is plausible that disease or cytokine-mediated changes in the expression and activity of OATP2B1 in the placenta could have potential adverse pregnancy outcomes. During development, placenta is the primary source of estrogen production, but requires fetal or maternal supply of the estrogen precursor, dehydroepiandrosterone (DHEA) or the sulfoconjugate, DHEA-S. OATP2B1 is involved in the uptake of DHEA-S and estrone-3-sulfate (E3S) from the fetal circulation into the placenta [33]. Grube et al. demonstrated that steroid hormone inhibitors of OATP could impose a pronounced decrease in OATP2B1-mediated uptake of E3S and DHEA-S [34]. Thus, the observed decrease in expression and activity of OATP2B1 may result in decreased placental estrogen production. Indeed, as compared to healthy pregnant women, a pronounced decrease in the production and expected increase in levels of estradiol is seen in SLE patients during their second and third trimester; a disease known to be associated with elevated levels of IL-23 [35]. Diminished availability of estrogen could negatively impact pregnancy outcomes, as estrogens support numerous processes that are essential for fetal growth and development and regulates the timing of parturition [36].

We also found that treatment of explants with IL-23 decreased the mRNA levels of OAT4. Significantly decreased placental mRNA expression of OAT4 was previously seen in pregnancies complicated with preeclampsia, a condition that is also associated with Th-17 activation and increased levels of IL-6, IL-17, and IL-23 [21,37]. OAT4 is involved in the uptake of the estriol precursor, 16a-hydroxy-DHEA-S, from the fetal compartment, and is considered to be its major transporter, thereby, contributing to more than 90% of placental estriol synthesis [38]. Therefore, decreased OAT4 could result in a further decrease in placental estrogen production. However, the clinical implications are unclear as the protein levels were not examined due to the limited amount of membrane proteins that could be isolated from the explants.

Th-17 associated cytokines were also found to alter the expression of the organic cation transporter, OCT3, as IL-23 alone or combined IL-23/IL-22/IL-17 treatment of explants, significantly increased the mRNA levels of OCT3. OCT3 is involved in the transport of fetal hormones, as well as several important neurotransmitters for fetal brain development [39,40]. Moreover, a number of clinically important drugs are substrates for OCT3, and therefore, can impact fetal drug exposure [41]. For example, OCT3 plays a pivotal role in facilitating maternal-to-fetal placental transport of metformin, a drug which is commonly used for the treatment of gestational diabetes [42]. Therefore, increased OCT3 expression could lead to increased fetal exposure to its substrates. Nevertheless, the clinical implications are unclear, as changes were only seen at the transcript but not the protein level.

Although the role of IL-17 has been recognized in several diseases, research has demonstrated that its impact is more pronounced in combination or in concert with other cytokines [43]. For example, IL-17A and IL-22 are believed to act in concert in autoimmune pathogenesis and IL-17 has been shown to act synergistically with TNF-α, in the induction of IL-6 in rheumatoid arthritis [44,45]. Therefore, placental explants were treated with a clinically relevant combination of IL-17A, IL-22, and IL-23, at concentrations seen in patients with autoimmune diseases [7]. Interestingly, we observed a downregulation of BCRP in explants treated with the cytokine combination. As BCRP is highly expressed in the placenta and plays an important role in the efflux of many clinically important drugs and environmental chemicals, downregulation could lead to increased fetal accumulation of potentially toxic compounds. Pronounced 45–60% reductions in the mRNA and protein expression of BCRP were previously seen in the placentas of pregnancies complicated by pre-eclampsia and chorioamnionitis [21,22]. Decreased protein and mRNA expression of BCRP has also been reported in bacterial endotoxin-treated, first-term placental explants, as well as in TNF-α-treated human primary cultured trophoblasts [17,27]. A corresponding increase in the cellular accumulation of mitoxantrone, a BCRP substrate, was seen in the TNF-α-treated trophoblasts [46]. Although we did not detect changes at the protein level, it is possible that exposure times of explants to the Th-17 cytokines were not sufficiently long to capture protein expression changes. However, our pilot studies indicated that longer culture periods of both the treated and control placental explants resulted in decreased viability and extremely high variability in the transcript and protein expression. Thus, further studies are needed to determine whether Th-17 cytokines and associated maternal diseases impair BCRP’s protective function in the placenta.

### Strengths and Limitations

A strength of this investigation lies in the examination of transporter regulation, using cultured placental explants of human origin. The heterogenous population and interaction of placental cells within the cultured explants, along with the functional expression of key inflammatory and hormonal signaling pathways, better emulates the in vivo situation. Therefore, placenta that is normally discarded after delivery, provides a great resource for clinically relevant ex vivo studies. However, they are susceptible to necrosis, which limits culture conditions and durations. Another limitation also stems from the large intra and inter-variability that can exist between different cotyledons of a single placenta or between different placentas.

## 5. Conclusions

In this study, we demonstrate that Th-17-associated cytokines cause a decrease in the expression and activity of OATP2B1 in cultured placental term explants. Alterations in the transcript levels of several other transporters were also seen. The balance between the uptake and efflux transporters in the placenta, determines the distribution of endogenous and exogenous substances between maternal and fetal compartments. As such, changes in the expression and activity of placental transporters can have clinically relevant impacts on fetal and maternal drug exposure, as well as fetal growth and development. As Th-17-associated cytokines are often elevated in pregnant women with several immune-related diseases, these findings have applicability to our understanding of maternal–fetal drug disposition. In addition, elevated Th-17 responses to SARS-COV-2 may also impact pregnant women during this pandemic. Therefore, it is important to understand the potential consequences of these changes on pregnancy and fetal outcomes.

## Figures and Tables

**Table 1:** Primer sequences used for qRT-PCR.

Gene	Sequence
*ABCG2* (BCRP)	Fw: 5′GGCCTTGCGATACTTTGAATC3′ Rv: 5′GAATCTCCATTAATGATGTCCA3′
*SLC22A11* (OAT4)	Fw: 5′ GGACCTGGAGAGCCAGAAATC3′ Rv: 5′GAGCGAGGTACTTTCCACAGTGA3′
*SLCO2B1* (OATP2B1)	Fw: 5′ACCCTCCTTCATGCTCATCCT3′ Rv: 5′ATGCCCACAGCCAAAGTCTT3′
*SLC22A3* (OCT3)	Fw: 5′TGCCAGAGACAGTGGATGATG3′ Rv: 5′TTTTCTTATTCCTGCCACATTTACAG3′
Cyclophilin A (PPIA)	Fw: 5′GCCCGTAGTGCTTCAGCTT3′ Rv: 5′GGAGATGGCACAGGAGGAA3′

## Data Availability

The data presented in this study are available on request from the corresponding author.
